# Systematic reviews, systematic error and the acquisition of clinical knowledge

**DOI:** 10.1186/1471-2288-10-53

**Published:** 2010-06-10

**Authors:** Steffen Mickenautsch

**Affiliations:** 1 Division of Public Oral Health, Faculty of Health Science, University of the Witwatersrand, 7 York Road, 2193 Parktown/Johannesburg, South Africa

## Abstract

**Background:**

Since its inception, evidence-based medicine and its application through systematic reviews, has been widely accepted. However, it has also been strongly criticised and resisted by some academic groups and clinicians. One of the main criticisms of evidence-based medicine is that it appears to claim to have unique access to absolute scientific truth and thus devalues and replaces other types of knowledge sources.

**Discussion:**

The various types of clinical knowledge sources are categorised on the basis of Kant's categories of knowledge acquisition, as being either 'analytic' or 'synthetic'. It is shown that these categories do not act in opposition but rather, depend upon each other. The unity of analysis and synthesis in knowledge acquisition is demonstrated during the process of systematic reviewing of clinical trials. Systematic reviews constitute comprehensive synthesis of clinical knowledge but depend upon plausible, analytical hypothesis development for the trials reviewed. The dangers of systematic error regarding the internal validity of acquired knowledge are highlighted on the basis of empirical evidence. It has been shown that the systematic review process reduces systematic error, thus ensuring high internal validity. It is argued that this process does not exclude other types of knowledge sources. Instead, amongst these other types it functions as an integrated element during the acquisition of clinical knowledge.

**Conclusions:**

The acquisition of clinical knowledge is based on interaction between analysis and synthesis. Systematic reviews provide the highest form of synthetic knowledge acquisition in terms of achieving internal validity of results. In that capacity it informs the analytic knowledge of the clinician but does not replace it.

## Background

Systematic reviews, in healthcare, have been described as providing objective overviews of all the evidence currently available on a particular topic of interest [1]. Such overviews cover clinical trials in order to establish where effects of healthcare are consistent and where they may vary. This is achieved through the use of explicit, systematic methods aimed at limiting systematic error (bias) and reducing the chance of effect [2]. Systematic reviews have been recommended as providing the best source of evidence to guide clinical decisions [3,4] and healthcare policy [5], and they receive twice as many citations as non-systematic reviews in peer-reviewed journals [5-7]. Furthermore, systematic reviews are increasingly utilized in appraising the evidence regarding the cost-effectiveness of interventions [8,9], the costs of guideline dissemination and implementation [10] or evidence from qualitative studies [11].

The use of systematic reviews for the appraisal of clinical studies has been introduced and promoted within the framework of evidence-based medicine (EBM). Sackett et al. recommended EBM as "the conscientious, explicit, and judicious use of current best evidence in making decisions about the care of individual patients" [12]. These authors defined the practice of evidence-based medicine as "integrating individual clinical expertise with the best available external clinical evidence from systematic research". They described best evidence as "clinically relevant research, often from the basic sciences of medicine, but especially from patient-centred clinical research into the accuracy and precision of diagnostic tests (including the clinical examination), the power of prognostic markers, and the efficacy and safety of therapeutic, rehabilitative, and preventive regimens" [12].

Since its inception, EBM has been widely accepted by academia, healthcare funders and healthcare providers. It has also been strongly criticised and resisted by some academic groups and clinicians. One of the main criticisms of EBM is that it claims to have unique access to absolute scientific truth, as gained for clinical therapy through randomized control trials (RCT) and subsequent systematic reviews of RCTs. The implication is that EBM claims, on this basis, the ability to exercise judgement (e.g. through appraisal of clinical studies during systematic reviews) and thus devalues and replaces knowledge sources of other types [13].

The types of knowledge sources allegedly threatened by EBM include: (i) the inferences of basic science used for prediction of clinical outcomes [14]; (ii) clinical judgement based on experience - often expressed in the form of single case studies and narrative reviews [15,16]; (iii) qualitative and observational research [16].

Further criticisms of EBM are that it produces population-based research results which are not applicable to individual patients and that research results from which any confounder impact is eliminated (i.e. through randomization and double blinding) can never wholly apply to particular individual situations faced by clinicians in their daily practice [15]. Critics of EBM argue that clinical trials ignore knowledge gained from basic science, in areas such as human physiology and diseases and pharmacology, from which valuable information about the effect of a particular drug or treatment can be inferred [14]. They hold that clinical judgement based on experience is more exact, because of its emphasis on individual cases rather than evidence derived from RCT [15]. As RCTs provide average estimates with confidence intervals from study groups instead of from single individual patients, their results remain allegedly non-applicable to daily clinical practice [15]. Therefore, it is argued, RCTs lack the necessary illustration of nuances of treatment that single-case reports provide [16]. Qualitative, as opposed to quantitative research, is seen to provide in-depth examination of small numbers of patients and is able, unlike hypothesis-driven quantitative EBM research, to provide information regarding the complexity (including psychological and social aspects) of a disease [16]. Qualitative research, it is further argued, has the capacity to explore the meanings that symptoms, consultations and treatments have for a patient -- aspects that EBM is accused of degrading or ignoring [16].

In response to such criticism, EBM promoters reply that sole reliance on basic science without clinical testing raises high uncertainties regarding treatment safety and efficiency [14]. Such uncertainties are based on the limits and incompleteness of basic scientific knowledge about the human body and its interaction with the environment [14]. In addition, it is reported that medical history confirms that therapeutic predictions based on sound basic science have, in many cases, been proven wrong after clinical testing [17]. One example is the well-cited case of Flecainide, which was used for treating supraventricular tachycardia. Only after clinical trials had been conducted was it found that it actually increased mortality in patients [18].

Reliance on clinical judgement based on experience can be misleading, owing to the unrecognised play of chance and the easy confusion of the natural history of the disease with the treatment effect [19]. For that reason, patients often get better or worse on their own, notwithstanding intervention [14]. A wide variation in clinicians' judgment has been observed in a group of 819 doctors from Australia and UK [20]. Only 55% correctly recognized the risks for ischaemic heart disease and just 6.7%, the risk of deep-vein-thrombosis. Traditional experience can also be a poor judge of the efficacy of treatments such as the widespread prophylactic removal of pathology-free impacted third molars to prevent cysts and tumours, resorption of second molars, caries and periodontal problems. In contrast, a systematic review found no evidence that this procedure offered clinical benefits [21]. Qualitative and observational study results are often tainted by systematic error and thus, lack the necessary internal validity that could allow any generalisation beyond the studied cases [14]. In terms of the criticism that EBM produces population-based research results that are not applicable to individual patients, EBM promoters respond that risks of disease, identified through population-based research, remain applicable to individual subjects. Once a causality has been detected, such causality will be as valid for individual patients in clinical practice as it is for subjects in the studied groups/populations [15]. Moreover, elimination of confounders through, for example, the randomization process in RCTs, does not render data irrelevant to individuals. Such data remains applicable to an individual patient, to the extent to which the patient shares the characteristics of the subjects studied in the RCT [22].

Against the background of such ongoing debate, this article aims to present a philosophical proposition regarding the acquisition of knowledge, which may help to clarify the relationship between the epistemological concepts that appear to underlie the different standpoints of EBM critics and promoters. It also aims to show how systematic reviews rely on the unity of analysis and synthesis in the process.

## Acquisition of knowledge

The German philosopher Immanuel Kant regarded experience as the direct encounter of a subject and an object, and knowledge as the judgment of such encounter [23,24]. Reflective judgment of experience could be either 'analytic' or 'synthetic'. While an analytic reflective judgment only asserts logical relations between concepts, a synthetic reflective judgment involves the assertion of real relationships between concepts and objects. Therefore, an analytical judgment of an experience recognises truth by virtue of conceptual meanings only, without depending further on external facts. An example of an analytical judgment is the statement: "Yellow is a colour". We know that this statement is correct. No additional evidence is needed because we know the meanings of the words "yellow" and "colour" [23,24].

In contrast, a synthetic judgment of experience recognises truth by virtue of conceptual meanings **and **external facts. Here, an example is the statement: "This table is yellow." Although we understand the meanings of the words "table" and "yellow", we still need to check whether the table is indeed yellow, thus requiring further evidence in order to accept that this statement is true [23,24].

The scientific method of analysis employs analytical reflective judgment. Analysis, according to the classical definition by Leipnitz, is a "process in which we begin with a given conclusion and seek principles by which we may demonstrate this conclusion" [25,26]. This means that causes are inferred from effects through assertion of logical relations between the two concepts and their relationship is used to develop plausible hypotheses [25,26]. During this process, care is taken to ensure that the resulting hypothesis does not contradict already existing knowledge. In clinical praxis this would mean that a doctor examines a patient, discovers symptoms and, on the basis of these and knowledge acquired from basic science and personal clinical experience, infers (diagnoses) a specific disease as the possible cause of such symptoms (effect). Similarly, in scientific research a possible/plausible hypothesis that could explain observations in line with current knowledge may be developed. However, a plausible hypothesis does not necessarily provide actual proof. Such proof may be found through the scientific method of synthesis. The classical definition of synthesis is "a process in which we begin from principles (= Cause) and proceed to build up conclusions" (= Effect) [25,26]. However, this is really only an inverted definition of analysis. It does not consider the need for outside facts and is thus limited to the inference of effects from known causes, (i.e. by inductive reasoning through Analogy or Teleology [25,27] in line with existing knowledge). The solution for this type of problem can be found in the work of Johann Gottlieb Fichte (often wrongly ascribed to Hegel). He defined synthesis as a result of the dialectic interaction/conflict between 'thesis' and 'antithesis' [28]. 'Thesis' represents a formulated idea or concept that can be, for example, an hypothesis developed through analysis. This hypothesis is then engaged by an opposing concept or fact, or external conditions that are not part of the initial hypothesis, created through experiment, scientific trial or other observations: the antithesis. Through this interaction/conflict, truths contained in the thesis and antithesis are reconciled at a higher level, thus forming synthesis. In turn, this synthesis constitutes a new thesis that is opposed by a new antithesis in a continuous process. Reflective judgement of the thesis in relation to the antithesis asserts real relationships between concepts and objects. Therefore, synthetic reflective judgment [23,24] is employed during the process of synthesis by thesis/antithesis [28]. One example is the 'extension for prevention' concept mentioned by GV Black (= Thesis) in relation to operative dentistry. It deals with the need to remove carious tooth tissue before restoring a tooth with amalgam, in order to prevent further caries progression [29]. An antithesis to this concept is the observation by Mertz-Fairhurst et al. that caries, after the sealing of retained carious tooth tissue, only progresses very slowly [30]. Frencken et al. reached a synthesis of both views, by introducing the atraumatic restorative treatment (ART) approach, on the basis of selective caries removal [31]. Selective caries removal according to the ART approach relies upon the removal of infected, soft tooth tissue, using only hand instruments. Affected, remineralisable carious tooth tissue is left behind and sealed with a biomimetic material. A recent systematic review with meta-analysis showed ART restorations to be clinically as successful as amalgam restorations placed according to GV Black's 'extension for prevention' concept [32]. Following Fichte's dialectic view of synthesis, scientists try to test the veracity of existing hypotheses through, for example, conducting clinical trials [27]. In this case, a null-hypothesis would form the thesis and the trial conditions, its antithesis. The result would be the synthesis in the form of rejection or acceptance of the null-hypothesis.

In this context the inference, extrapolation (projection from basic science) and application of clinical judgement based on experience are analytic; while synthesis is represented in the conduct of clinical case studies, qualitative-, observational- and randomized control trials, and in systematic reviews with meta-analysis.

## Systematic error

Systematic error constitutes any factor in the knowledge acquisition process that systematically diverts its outcomes away from true values [33]. Systematic error, therefore, limits the internal validity of acquired knowledge. Internal validity depends upon the linking together, apart from random error, of an inferred or investigated cause and effect; thus ensuring causality [34]. With regard to analytic knowledge acquisition, the problems of (i) inferring from basic science and (ii) applying clinical judgement based on experience alone have been highlighted above [14,17]. With regard to synthetic knowledge acquisition, a range of systematic errors has been identified: selection-, performance-/detection-, and attrition bias [34]. In order to limit the influence of systematic error on clinical trials, the methodological interventions: randomization (random sequence allocation and allocation concealment), blinding and intention-to-treat analysis have been proposed for each type of bias, respectively [34].

Empirical evidence from meta-epidemiological studies indicates that without the application of methodological bias-controlling measures in clinical trials, a systematic error effect may manifest itself in the form of a substantial over-estimation of results. Trials that investigate subjective outcome measures are especially at risk. The level of over-estimation associated with attrition bias (lack of intention-to-treat analysis) can reach up to 25% [35]. The lack of adequate randomization (through sequence allocation and allocation concealment) and blinding (thus minimizing Selection- and Performance-/Detection bias, respectively) may reach above 50% [36]. This means that if a study claims a 20% lower relative risk (RR 0.80) for a new treatment, as compared to a control under a condition of a 50% overestimation, the actual result of the observed treatment effect would be a 20% increased risk (RR 1.20) for the patient. Thus, it would be the complete opposite of the initial claim. Such high percentages of over-estimation due to bias may therefore lead to situations where ineffective treatment procedures are presented as effective.

The empirical evidence regarding the danger of systematic error suggests that inclusion of bias-controlling measures; such as randomization, blinding and attrition control, into the study design of clinical trials is justified. It also provides the justification for judging the internal validity of clinical trials according to how well bias-controlling measures are implemented in their study designs; i.e. in line with an evidence-hierarchy [37]. Assessment of clinical trials according to their internal validity is part of the systematic review process.

## Systematic review

Systematic reviews are defined, according to the Cochrane collaboration, as scientific literature reviews aimed at answering clearly formulated questions by use of systematic and explicit methods for identifying, selecting, and critically appraising relevant research, and for collecting and analysing data from the literature included in the review [38]. During a systematic review, meta-analysis may be used as a statistical tool for analysing and summarising the results of the included studies [39]. In order to fulfil this function, a systematic review should: (i) present a synthesis of the acquired knowledge regarding one particular clinical question derived from all relevant studies that are identifiable at one point in time, (ii) identify the level of internal validity and the subsequent potential systematic error risk associated with the acquired knowledge and (iii) provide recommendations for improving any identified shortcoming related to internal validity, for further research. Owing to continued further research, systematic reviews should also provide continued updates of their synthesis.

In order to achieve its objectives, a systematic review includes (i) a systematic search for studies from all known and relevant information sources; (ii) the selection of studies with highest internal validity -- or if not many studies can be found, the sub-grouping of available trials in line with their various internal validity strengths; (iii) quality assessment of studies in line with internal validity criteria and, if possible, (iv) meta-analysis of the combined study data.

Through this process, systematic reviews provide the most comprehensive answers to clinical questions, with least possible systematic error. Such high internal validity provides a basis for the external validity of results. External validity describes how well results can be generalised and are applicable to other circumstances [34]. Evidence that is free of systematic error appears to be more likely to remain correct, even under changing circumstances, than results that carry a high risk of over-estimation. However, although external validity can only be possible on the basis of good internal validity [34], good internal validity of evidence from systematic reviews on its own has been shown to provide no absolute guarantee of good external validity. A case study [40], during which the conduct and management of a systematic review of studies concerning interventions for reducing substance misuse in young children was observed, noted the exclusion of review articles that did not follow a systematic methodology but contained explicit considerations of wider environmental factors impacting upon substance misuse. This study reported that the subsequent guideline development process resolved to ad-hoc inferences regarding the application of the systematic review results, due to its lack of external validity focus [40]. Apart from future systematic reviews with more emphasis on categories of external validity, qualitative research may add important information regarding the external validity of evidence, by investigating the complexity of, for example, the psychological and social aspects of disease [16]. Single case reports may indeed provide the necessary illustration of nuances during the judicious use of current best evidence [16]. For example, a case report [41] that informed on aspects of implementation and patients' response to atraumatic restorative treatment (ART) in an oral healthcare service provided important insights concerning the external validity of ART results that were established through a relevant systematic review [33]. Through systematic reviews focussing on high internal validity, analytical clinical judgment becomes more informed [12]. This implies that synthesis informs analysis and is not in opposition to it as the debate between EBM promoters and critics seems to suggest. Instead, both analysis and synthesis exist in unity.

## Analysis and synthesis unity

**Figure 1:**
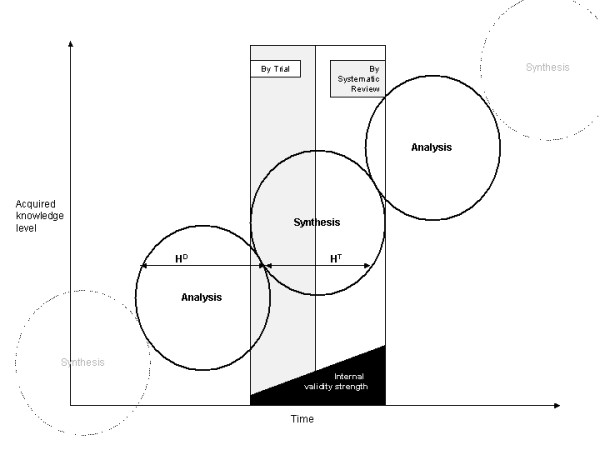
**Analysis, Synthesis unity**. H^D ^= Hypothesis development. H^T ^= Hypothesis testing

The unity of analysis and synthesis is demonstrated in the suggested model (Figure 1). Analytical knowledge derived through projection from basic science, as well as from experiences, forms the basis for a plausible hypothesis (H). It has been suggested that any empirical test results are meaningless if the tested hypothesis violates principles of basic science [14]. For example, evidence from RCTs supporting the claim of homeopathic remedies to be effective beyond the placebo effect would be seriously doubted, as knowledge derived from basic science does not provide an explanation of how highly diluted homeopathic solutions can contain any active ingredient capable of causing any observed significant (p < 0.05) treatment effect [42]. This implies that analysis justifies synthesis. Therefore, as shown above on hand of a plausible hypothesis development [25], sources of "other knowledge" on an analytical basis are extremely important in hypothesis development (H^D^).

The development of a plausible hypothesis needs to be followed by hypothesis testing (H^T^). Such testing has to take into consideration the empirical evidence [35,36] for the negative impact of systematic error. This requires a focus on inclusion into the study design of clinical trials, of bias-controlling measures: randomization, blinding and attrition control. Results of clinical trials that utilize such measures, like RCTs can therefore be considered to have higher internal validity in terms of hypothesis testing. Synthesis by trial is obtained through engagement of the hypothesis (= Thesis) with the rigor of the clinical trial methodology (= Antithesis). However, the knowledge acquired through synthesis by one single trial stands isolated from the results of other trials with similar focus. A systematic review with meta-analysis achieves unification of isolated trial results and thus, can provide a more comprehensive answer to clinical questions than one single trial can. For example, the pooled results of one meta-analysis that included 31 randomized control trials indicated a reduction of risk of recurrence of breast cancer after chemotherapy, in contrast to no chemotherapy, while the individual result of each trial was inconclusive [43]. The synthesis from systematic reviews that include meta-analysis is based (in direct proportion to the sample size of each trial) on the weighted comparison between combined data of conventional treatments as control (= Thesis), with the combined data of newly developed (test-) interventions (= Antithesis). During this process bias-controlling measures, such as the selection of trials with high internal validity (e.g. RCT for therapy related topics), and quality assessment of trials, are utilized.

Through synthesis by systematic review, a comprehensive answer to clinical questions is achieved, with least possible systematic error and with high internal validity. On this basis, the analytic knowledge of the clinician is informed. According to a model by Glaszion and Irwig [44], systematic review results of RCTs would provide a doctor with, for example, information about the net benefit of Warfarin treatment for a patient with fibrillation and the risk of thromboembolic stroke. A systematic review of cohort studies would provide information regarding the potential harm of such treatment (e.g. induction of intracranial bleeding by Warfarin). This evidence would also reveal that the benefit of Warfarin increases along with the increase in risk for thromboembolic stroke and that the danger of for example, bleeding, remains constant. Armed with such information, the doctor would examine his patient for signs of major risk factors such as high blood pressure or previous thromboembolism. The doctor could then, on the basis of the evidence, be able to judge that in absence of any major risk factors, the benefit of Warfarin treatment would be outweighed by its potential harm and might thus decide against treating the patient with Warfarin. From this process new analytical knowledge is formed and clinical judgment altered and updated and, in time, clinical experience on a higher level of acquired knowledge is developed. Such clinical experience in turn provides the analytical basis for future hypothesis development in line with basic science, thus forming a repeated interaction between analysis and synthesis. The repeated interaction results in the continued acquisition of clinical knowledge on higher levels over time.

The acquisition of clinical knowledge is based on the interaction between analysis and synthesis. It is erroneous to judge one as being superior to the other. Systematic reviews provide the highest form of synthetic knowledge acquisition in terms of achieving internal validity of results. However, this should not imply that systematic reviews are generally superior to other forms of knowledge or can replace, for example, the function of qualitative research results, particularly in relation to aspects of external validity and clinical judgment regarding the care of individual patients. On the other hand, analytical clinical judgment that is not informed by high internal validity synthesis becomes in time obsolete for patient treatment and faces the danger of being affected by systematic error.

## Competing interests

The author contributes to the conduct and publication of systematic reviews concerned with topics related to Minimum Intervention (MI) in dentistry.

## Authors' contributions

SM developed the concept and outline and wrote this paper.

## Pre-publication history

The pre-publication history for this paper can be accessed here:

http://www.biomedcentral.com/1471-2288/10/53/prepub

## References

[B1] Altmann DG What randomized trials and systematic reviews can offer decision makers Horm Res 1999 51 36 43 10.1159/000053134 10393490

[B2] Higgins JPT Green S (Eds) Cochrane handbook for systematic reviews of interventions 4.2.6 [updated October 2007] 2006 Chichester, UK: John Wiley & Sons, Ltd

[B3] Milrow CD Cook DJ Davidoff F Systematic reviews: critical links in the great chain of evidence Ann Intern Med 1997 126 389 391 9054284 10.7326/0003-4819-126-5-199703010-00008

[B4] Lavis JN Posada FB Haines A Osei E Use of research to inform public policymaking Lancet 2004 364 1615 1621 10.1016/S0140-6736(04)17317-0 15519634

[B5] Bero LA Jadad AR How consumers and policymakers can use systematic reviews fro decision making Ann Intern Med 1997 127 37 42 9214251 10.7326/0003-4819-127-1-199707010-00007

[B6] Bhandari M Montori VM Devereaux PJ Wilczynski NL Morgan D Haynes RB The Hedges Team Doubling the impact: publication of systematic review articles in orthopaedic journals J Bone Joint Surg Am 2004 86-A 1012 1016 15118046

[B7] Montori VM Wilczynski NL Morgan D Haynes RB Hedges Team Systematic reviews: a cross-sectional study of location and citation counts BMC Med 2003 1 2 10.1186/1741-7015-1-2 14633274 PMC281591

[B8] Oxman AD Fretheim A Lavis JN Lewin S SUPPORT Tools for evidence-informed health Policymaking (STP). 12. Finding and using research evidence about resource use and costs Health Res Policy Syst 2009 7 Suppl 1 S12 10.1186/1478-4505-7-S1-S12 20018102 PMC3271823

[B9] Renfrew MJ Craig D Dyson L McCormick F Rice S King SE Misso K Stenhouse E Williams AF Breastfeeding promotion for infants in neonatal units: a systematic review and economic analysis Health Technol Assess 2009 13 1 iv 19728934 10.3310/hta13400

[B10] Grimshaw JM Thomas RE MacLennan G Fraser C Ramsay CR Vale L Whitty P Eccles MP Matowe L Shirran L Effectiveness and efficiency of guideline dissemination and implementation strategies Health Technol Assess 2004 8 iii 72 14960256 10.3310/hta8060

[B11] Mays N Pope C Popay J Systematically reviewing qualitative and quantitative evidence to inform management and policy-making in the health field J Health Serv Res Policy 2005 10 Suppl 1 6 20 10.1258/1355819054308576 16053580

[B12] Sackett DL Rosenberg WMC Gray JAM Haynes RB Richardson WS Evidence based medicine: what it is and what it isn't: It's about integrating individual clinical expertise and the best external evidence BMJ 1996 312 71 72 8555924 10.1136/bmj.312.7023.71 PMC2349778

[B13] Buetow S Beyond evidence-based medicine: bridge-building a medicine of meaning J Evaluation Clin Pract 2002 8 103 108 10.1046/j.1365-2753.2002.00340.x 12180358

[B14] Sehon SR Stanley DE A philosophical analysis of the evidence-based medicine debate BMC Health Services Res 2003 3 14 10.1186/1472-6963-3-14 PMC169187 12873351

[B15] Parker M False dichotomies: EBM, clinical freedom, and the art of medicine Med Humanities 2005 31 23 30 10.1136/jmh.2004.000195 16167410

[B16] Williams DDR Garner J The case against 'the evidence': a different perspective on evidence-based medicine Br J Psychiatry 2002 180 8 12 10.1192/bjp.180.1.8 11772844

[B17] Doust J Del Marc C Why do doctors use treatments that do not work? BMJ 2004 328 474 10.1136/bmj.328.7438.474 14988163 PMC351829

[B18] Echt DS Liebson PR Mitchell LB Peters RW Obias-Manno D Barker AH Arensberg D Baker A Friedman L Greene HL Mortality and morbidity in patients receiving encainide, flecainide, or placebo. The Cardiac Arrhythmia Suppression Trial N Engl J Med 1991 324 781 788 1900101 10.1056/NEJM199103213241201

[B19] Reilly BM Hart A Evans AT Part II. Evidence-based medicine: a passing fancy or the future of primary care? Dis Mon 1998 44 370 399 10.1016/S0011-5029(98)90006-2 9735941

[B20] Attia JR Nair BR Sibbritt DW Ewald BD Paget NS Wellard RF Patterson L Heller RF Generating pre-test probabilities: a neglected area in clinical decision making Med J Aust 2004 180 449 454 15115422 10.5694/j.1326-5377.2004.tb06020.x

[B21] Song F Landes DP Glenny AM Sheldon TA Prophylactic removal of impacted third molars: an assessment of published reviews Br Dent J 1997 182 339 346 9175290 10.1038/sj.bdj.4809378

[B22] Parker M Whither our art? Clinical wisdom and evidence based medicine Med Health Care Philos 2002 5 275 276 10.1023/A:1021116516342 12517035

[B23] Kant I The critique of pure reason. Trans. by P. Guyer and A.W. Wood 1781 1998 Cambridge University Press

[B24] Palmquist S Kant's System of Perspectives: An architectonic interpretation of the Critical philosophy 1993 University Press of America

[B25] Ritchey T Analysis and Synthesis. On Scientific Method - Based on a Study by Bernhard Riemann Systems Res 1991 8 21 41

[B26] Leipnitz GW Loemker L Philosophical Papers and Letters 1956 Chicago, USA: University Press

[B27] Djulbegovic B Guyatt GH Ashcroft RE Epistemologic inquiries in evidence-based medicine Cancer Control 2009 16 158 168 19337202 10.1177/107327480901600208

[B28] Fichte JG Attempt at a critique of all revelations. Trans. by G. Green 1792/793 1978 Cambridge University Press

[B29] Black GV A work on operative dentistry: The technical procedures in filling teeth 1917 Chicago. Medico- Dental Publishing Company19979543

[B30] Mertz-Fairhurst EJ Schuster GS Williams JE Fairhurst CW Clinical progress of sealed and unsealed caries. Part II: Standardized radiographs and clinical observations J Prosthet Dent 1979 42 633 637 10.1016/0022-3913(79)90193-8 292775

[B31] Frencken JE Pilot T Songpaisan Y Phantumvanit P Atraumatic restorative treatment (ART): rationale, technique, and development J Public Health Dent 1996 56 135 140 10.1111/j.1752-7325.1996.tb02423.x 8915958

[B32] Mickenautsch S Yengopal V Banerjee A Clinical application of GIC: Atraumatic restorative treatment versus amalgam restoration longevity: a systematic review Clin Oral Investig 2009 DOI 10.1007/s00784-009-0335-8 19688227 10.1007/s00784-009-0335-8

[B33] Murphy EA The logic of medicine 1976 Baltimore: John Hopkins University Press

[B34] Jüni P Altman DG Egger M Systematic reviews in health care. Assessing the quality of controlled clinical trials BMJ 2001 323 42 46 10.1136/bmj.323.7303.42 11440947 PMC1120670

[B35] Stewart LA Parmar MK Meta-analysis of the literature or of individual patient data: is there a difference? Lancet 1993 341 418 422 10.1016/0140-6736(93)93004-K 8094183

[B36] Egger M Jüni P Bartlett C Holenstein F Sterne J How important are comprehensive literature searches and the assessment of trial quality in systematic reviews? Empirical study Health Technology Assessment 2003 7 1 12583822

[B37] Sutherland SE Evidence-based dentistry: Part IV. Research design and level of evidence J Can Dent Assoc 2001 67 375 378 11468093

[B38] The Cochrane collaboration What are Cochrane Reviews? http://www.cochrane.org/cochrane-reviews [accessed 01.06.2010]

[B39] Green S, Higgins J Glossary. Cochrane Handbook for Systematic Reviews of Interventions 2005 http://www.cochrane.org/resources/handbook/[accessed 27.04.2010]

[B40] Pearson M Coomber R The challenge of external validity in policy-relevant systematic reviews: a case study from the field of substance misuse Addiction 2010 105 136 145 10.1111/j.1360-0443.2009.02713.x 19804458

[B41] Mickenautsch S Rudolph MJ Ogunbodede EO Frencken JE The impact of the ART approach on the treatment profile in a mobile dental system (MDS) in South Africa Int Dent J 1999 49 132 138 10858745 10.1002/j.1875-595x.1999.tb00897.x

[B42] Linde K Clausius N Ramirez G Melchart D Eitel F Hedges LV Jonas WB Are the clinical effects of homeopathy placebo effects? A meta-analysis of placebo-controlled trials Lancet 1997 350 834 843 10.1016/S0140-6736(97)02293-9 9310601

[B43] Systemic treatment of early breast cancer by hormonal, cytotoxic, or immune therapy. 133 randomised trials involving 31,000 recurrences and 24,000 deaths among 75,000 women. Early Breast Cancer Trialists' Collaborative Group Lancet 1992 339 71 85 1345869

[B44] Glasziou PP Irwig LM An evidence based approach to individualising treatment BMJ 1995 311 1356 1359 7496291 10.1136/bmj.311.7016.1356 PMC2551234

